# Identification of crucial genes and metabolites regulating the eggshell brownness in chicken

**DOI:** 10.1186/s12864-022-08987-7

**Published:** 2022-11-21

**Authors:** Jing Yang, Zhiqiong Mao, Xiqiong Wang, Jingjie Zhuang, Sijia Gong, Zhouyang Gao, Guiyun Xu, Ning Yang, Congjiao Sun

**Affiliations:** 1 National Engineering Laboratory for Animal Breeding and Key Laboratory of Animal Genetics, Breeding and Reproduction, Ministry of Agriculture and Rural Affairs, China Agricultural University, Beijing, 100193 China; 2 Beinongda Technology Co,.Ltd, Beijing, 100083 China

**Keywords:** Protoporphyrin IX, Brown eggshell, δ-aminolevulinate synthase 1, Shell gland, Oviposition

## Abstract

**Background:**

Protoporphyrin IX (Pp IX) is the primary pigment for brown eggshells. However, the regulatory mechanisms directing Pp IX synthesis, transport, and genetic regulation during eggshell calcification in chickens remain obscure. In this study, we investigated the mechanism of brown eggshell formation at different times following oviposition, using White Leghorn hens (WS group), Rhode Island Red light brown eggshell line hens (LBS group) and Rhode Island Red dark brown eggshell line hens (DBS group).

**Results:**

At 4, 16 and 22 h following oviposition, Pp IX concentrations in LBS and DBS groups were significantly higher in shell glands than in liver (*P* < 0.05). Pp IX concentrations in shell glands of LBS and DBS groups at 16 and 22 h following oviposition were significantly higher than WS group (*P* < 0.05). In comparative transcriptome analysis, δ-aminolevulinate synthase 1 (*ALAS1*), solute carrier family 25 member 38 (*SLC25A38*), ATP binding cassette subfamily G member 2 (*ABCG2*) and feline leukemia virus subgroup C cellular receptor 1 (*FLVCR1*), which were associated with Pp IX synthesis, were identified as differentially expressed genes (DEGs). RT-qPCR results showed that the expression level of *ALAS1* in shell glands was significantly higher in DBS group than in WS group at 16 and 22 h following oviposition (*P* < 0.05). In addition, four single nucleotide polymorphisms (SNPs) in *ALAS1* gene that were significantly associated with eggshell brownness were identified. By identifying the differential metabolites in LBS and DBS groups, we found 11-hydroxy-E4-neuroprostane in shell glands and 15-dehydro-prostaglandin E1(1-) and prostaglandin G2 2-glyceryl ester in uterine fluid were related to eggshell pigment secretion.

**Conclusions:**

In this study, the regulatory mechanisms of eggshell brownness were studied comprehensively by different eggshell color and time following oviposition. Results show that Pp IX is synthesized de novo and stored in shell gland, and *ALAS1* is a key gene regulating Pp IX synthesis in the shell gland. We found three transporters in Pp IX pathway and three metabolites in shell glands and uterine fluid that may influence eggshell browning.

**Supplementary Information:**

The online version contains supplementary material available at 10.1186/s12864-022-08987-7.

## Background

Eggshell color is one of the most visible characteristics of an egg, and acts as a direct reference trait for consumers to evaluate egg quality, particularly for brown eggs. Eggshell color has a certain effect on eggshell structure, but there was a minor correlation between eggshell color and internal egg quality [1–4]. Brown eggshells contain abundant Protoporphyrin IX (Pp IX) [5], the concentration of which determines the brownness of the eggshell [6, 7]. The deposition of Pp IX in the eggshell coincides with calcification. Approximately 5.5 h following oviposition (the expulsion of an egg into the external environment), the next egg reaches the red isthmus of the oviduct and begins to form the papillary layer of the eggshell [8], indicating the beginning of new eggshell formation. At this phase, pigment particles can be observed in the lumens of epithelial cells of the shell gland [9]. The pigment content of the shell gland steadily increases until 20 h following oviposition, although the eggshell remains white during this time [10]. The pigment accumulated in the epithelial cells of the shell gland begins to move to the uterine fluid 2–3 h prior to subsequent oviposition. The pigment is then progressively deposited on the surface of the eggshell [11], with maximum deposition rates occurring 1–2 h prior to oviposition [10].

Pp IX is a direct precursor of heme, following the same metabolic pathway [12]. Pp IX is synthesized via seven enzymatic reactions, in which several synthases and transporters are believed to be involved. In nonerythroid cells, the first of seven steps in Pp IX synthesis, mediated by *ALAS1*, is the condensation of succinyl coenzyme A and glycine to generate δ-aminolevulinic acid (ALA) in the mitochondrial matrix, which is a rate-limiting step during the synthesis [13–15]. The synthesized Pp IX and iron ions are catalyzed by ferrochelatase (*FECH*) to synthesize heme, a reaction that is reversible. Even though Pp IX’s biosynthesis pathway is known, there are differing hypotheses on its synthesis site and precursor origin. Many studies suggest that Pp IX is synthesized de novo and accumulates in epithelial cells of the shell gland [16, 17]. The amount of pigment particles in the epithelial cells of the shell gland has been shown to increase in accordance with the process of eggshell formation until the protective membrane is formed [9]. In one study, Li et al. [18] compared Pp IX contents in the shell gland, serum, bile and excreta of chickens lay dark and light brown shelled eggs at 6 h following oviposition, and found significant differences only within the shell gland. The expression level of *ALAS1* in the shell gland of hens laying eggs with dark brown eggshells was significantly higher than in those laying eggs with light brown eggshells. The expression levels of genes involved in Pp IX synthesis in shell gland at 2, 5, 15, and 22.5 h following oviposition were also found to be related to eggshell formation, providing strong evidence that Pp IX is synthesized de novo in the shell gland [19]. However, it has also been proposed that Pp IX in shell glands is derived from free Pp IX in the blood [20, 21], or from conversion of heme, which is released by aging red blood cells in the blood [22].

Numerous studies have shown that eggshell brownness is regulated by synthases and transporters in the Pp IX/heme synthesis pathway. *ALAS1* is the target gene for the regulation of Pp IX concentration in eggshells [19, 23]. *ABCG2* has been shown to regulate ALA-mediated Pp IX levels by exporting Pp IX from the mitochondria to the cytoplasm [24]. Molecular experiments also show that coproporphyrinogen oxidase (*CPOX*) is expressed at higher levels in brown eggshell hens, but *FECH* is expressed at higher levels in white and pink eggshell hens [25]. Additionally, an iTRAQ-based quantitative proteomic analysis by Li et al. [26] revealed that proteins which transport Pp IX and iron into the mitochondrial matrix where heme is synthesized were upregulated and proteins that promote extracellular transport of Pp IX were downregulated in the shell glands of hens laying light brown eggs compared to hens laying dark brown eggs.

Existing studies on the mechanism of eggshell brownness, however, have only considered eggshell brownness from the perspective of the brownness intensity or time following oviposition, without comprehensive consideration of these two factors. Here, we held that combining these two perspectives to reveal the intrinsic driving impact of eggshell brownness was indeed necessary and of great value. Therefore, in the current study, hens with white, light brown and dark brown eggshells were utilized to explore the synthesis sites of Pp IX, and regulatory mechanism of brown eggshell formation at different stages of calcification. These findings may help shed light on the mechanism underlying brown pigmentation in eggshell.

## Results

### Influence of eggshell brownness selection on egg quality

To investigate the impact of eggshell brownness on egg quality, we measured egg weight and eggshell-related traits of light brown eggshell (LBS) line and dark brown eggshell (DBS) line, which have been bidirectionally selected for six generations according to their eggshell brownness. The results demonstrated that the DBS line had significantly darker eggshells than the LBS line (*P* < 0.05, Table 1). The egg weight, eggshell strength, and eggshell weight of the LBS line eggs were significantly higher than those of the DBS line eggs (*P* < 0.05, Table 1), however, there was no significant difference in eggshell thickness and eggshell proportion.

**Table 1:** Comparison of egg quality between LBS and DBS line eggs

Variable	Line	
	LBS	DBS
N^c^	150	150
Egg weight (g)	58.71 ± 4.87^a^	56.99 ± 3.80^b^
L	71.26 ± 3.84^a^	63.16 ± 3.43^b^
a	12.15 ± 2.53^a^	16.87 ± 1.85^b^
b	24.96 ± 3.03^a^	29.36 ± 1.62^b^
Eggshell strength (kg/cm^2^)	3.612 ± 0.944^a^	3.317 ± 0.674^b^
Eggshell thickness (mm)	0.344 ± 0.027	0.339 ± 0.023
Eggshell weight (g)	6.20 ± 0.53^a^	5.98 ± 0.45^b^
Eggshell proportion (%)	10.61 ± 0.77	10.49 ± 0.68

Data were expressed as mean ± standard deviation. Different superscript letters ^a, b^ across a row indicate significant differences

^c^One egg each from 150 hens from each of the LBS and DBS lines was analyzed

### Pp IX Concentrations in liver and shell glands

**Fig. 1:**
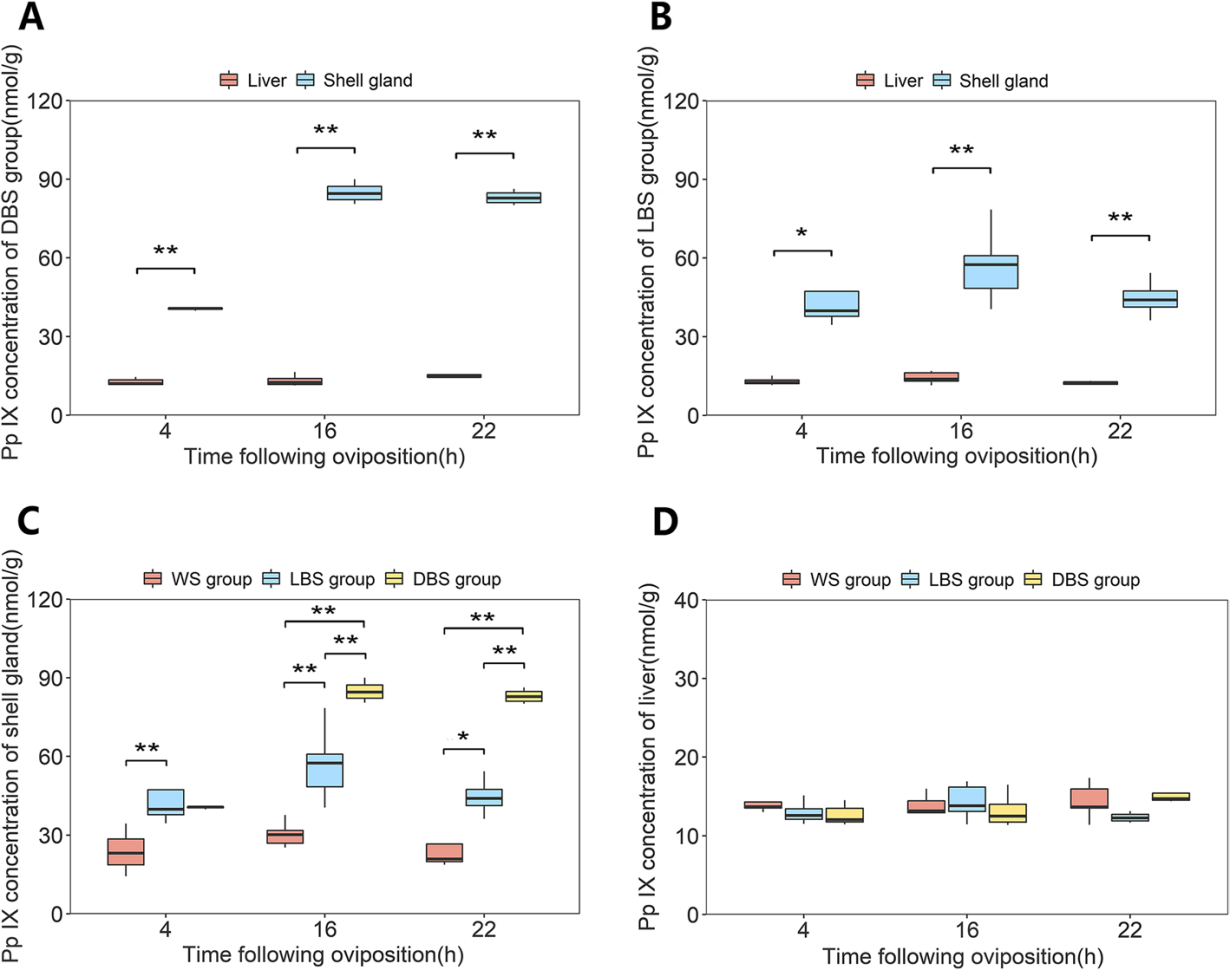
Concentrations of Pp IX in liver and the mucosal layer of shell glands of hens with different eggshell colors. **A** Pp IX concentrations in the liver and the mucosal layer of shell gland at 4H, 16H and 22H in the DBS group. **B** Pp IX concentrations in the liver and the mucosal layer of shell gland at 4H, 16H and 22H in the LBS group. **C** Pp IX concentrations in the mucosal layer of shell gland of the WS, LBS and DBS groups at 4H, 16H and 22H. **D** Pp IX concentrations in the liver of the WS, LBS and DBS groups at 4H, 16H and 22H. Data expressed as mean ± SD. **P < 0.01, *P < 0.05

Eggshell coloring occurs in accordance with eggshell calcification. At 4 h following oviposition (4H), egg has not yet entered the shell gland, hence eggshell calcification does not begin. At 16 h following oviposition (16H), the egg undergoes a rapid calcification process in the shell gland, but pigment deposition does not initiate at this time. At 22 h following oviposition (22H), eggshell calcification reaches its terminal stage, and pigment deposition is extensive within shell gland (Additional file 1: Fig. S1). To determine the synthesis and storage site of Pp IX, we measured Pp IX concentrations in liver and shell glands at 4H, 16H and 22H. The results showed that the concentration of Pp IX in the shell gland was significantly higher than that in the liver at all three time points in both the LBS and DBS group (*P* < 0.01 or *P* < 0.05, Fig. 1A and B). To clarify the synthesis pattern, white leghorn chickens that lay eggs with white eggshells (WS) were used as a control group. Figure 1C shows that the concentration of Pp IX in shell glands of the WS group was very low at all three time points (23.80–0.35 nmol/g). Nevertheless, the concentration of Pp IX in the shell glands of LBS and DBS groups reached their highest level at 16H (*P* < 0.05), especially in the DBS group (84.92 nmol/g). In the liver, there was no significant difference in the concentration of Pp IX among WS, LBS, and DBS groups at any of the three time points (*P* > 0.05, Fig. 1D).

### Identification of differentially expressed genes from RNA-sequencing data

**Fig. 2:**
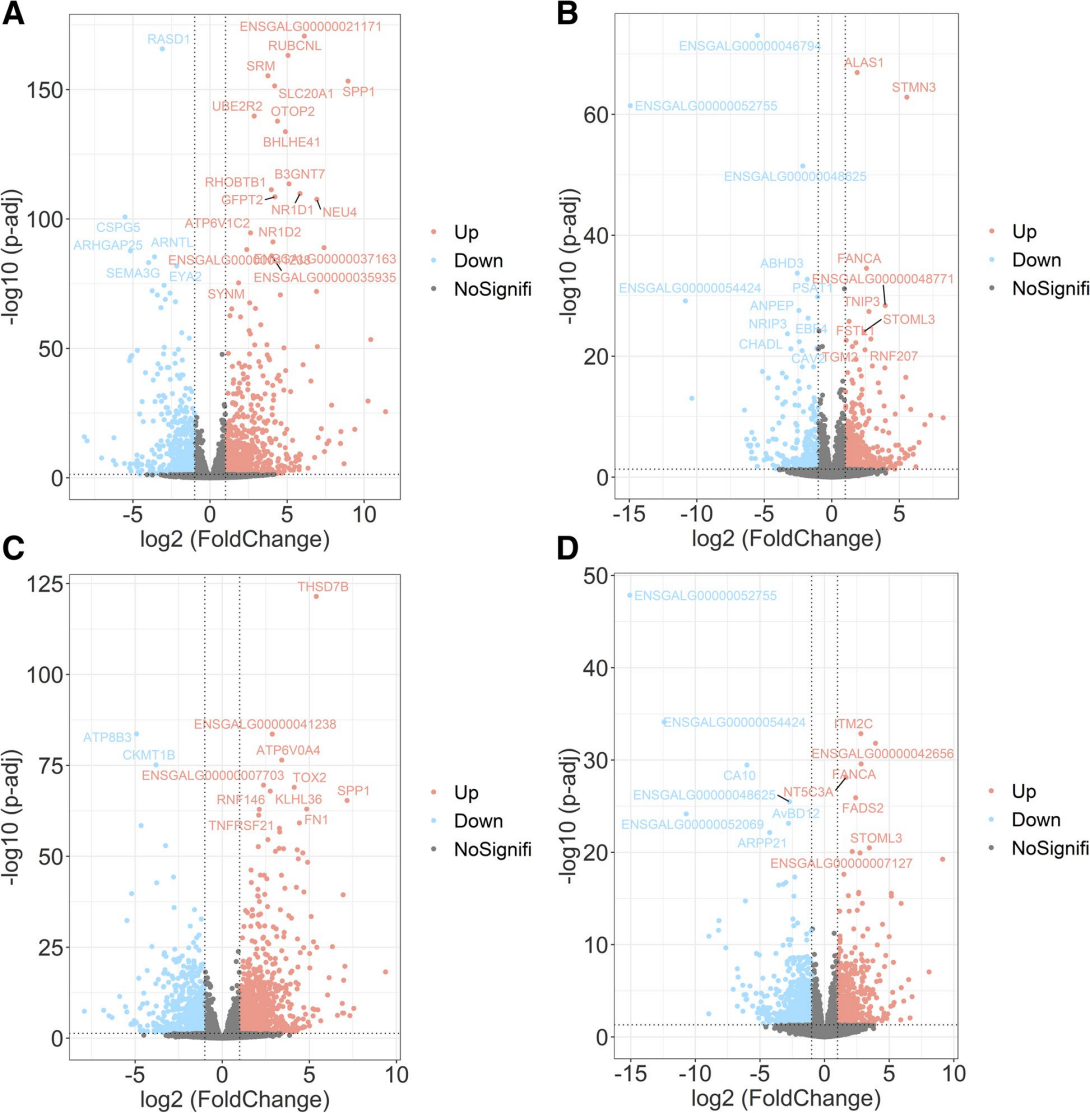
Volcano diagram of DEGs of four comparison groups. **A** DEGs between 16H and 4H of DBS group, based on 4H; **B** DEGs between 22H and 4H of DBS group, based on 4H; **C** DEGs between DBS and WS groups at 16H, based on WS group; **D** DEGs between DBS and WS groups at 22H, based on WS group. Genes that are significantly upregulated are shown in red, genes that are significantly downregulated in blue, and genes that are not significantly different are shown in gray

**Fig. 3:**
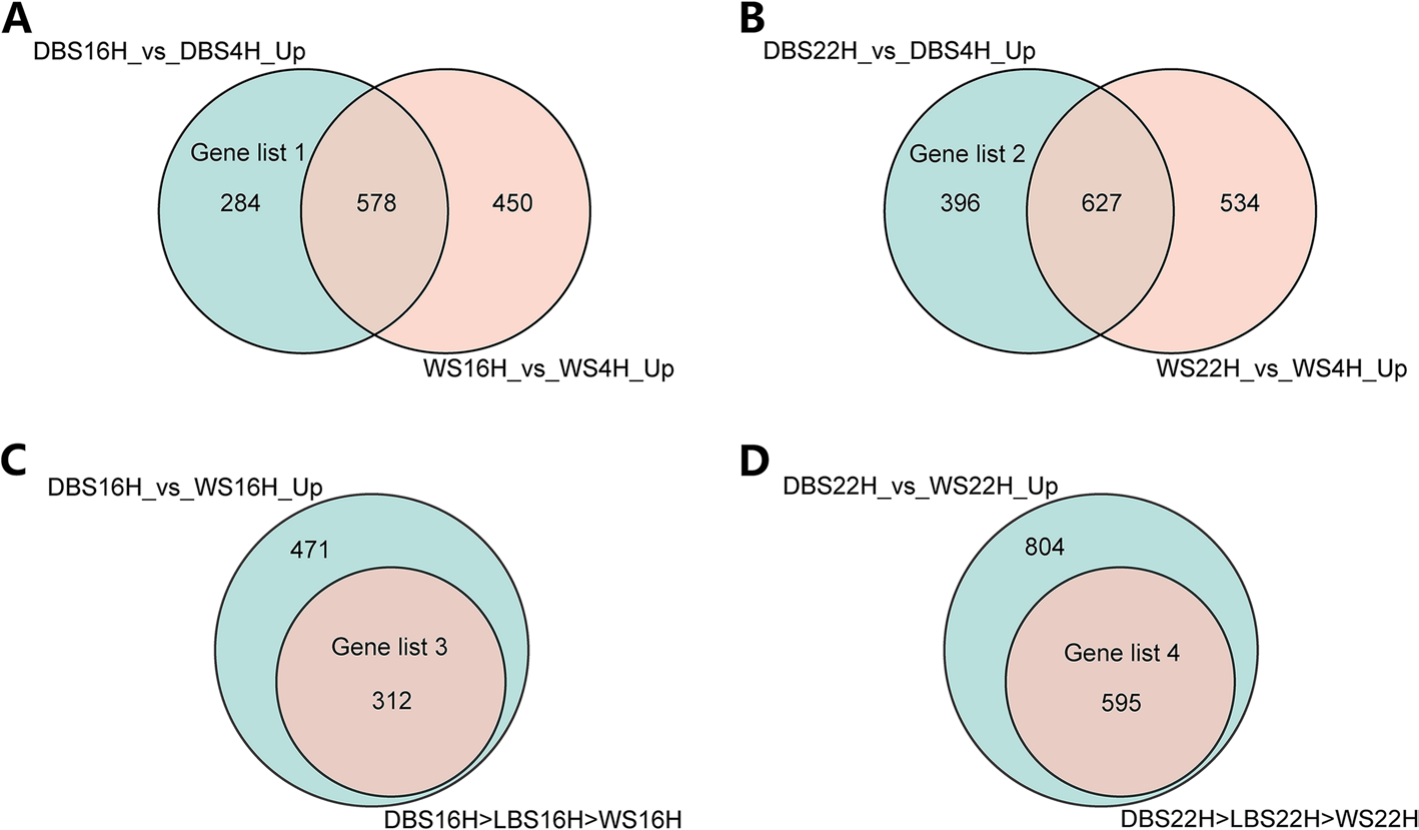
Diagram of Gene lists 1–4. **A** Gene list 1. **B** Gene list 2. **C** Gene list 3. **D** Gene list 4. DBS16H_vs_DBS4H_Up, significantly upregulated genes at 16H in the DBS group compare to 4H; WS16H_vs_WS4H_Up, significantly upregulated genes at 16H in the WS group compare to 4H; DBS22H_vs_DBS4H_Up, significantly upregulated genes at 22H in the DBS group compare to 4H; WS22H_vs_WS4H_Up, significantly upregulated genes at 22H in the WS group compare to 4H; DBS16H_vs_WS16H_Up, significantly upregulated genes in the DBS group at 16H compare to the WS group; DBS16H > LBS16H > WS16H, genes whose expression level at 16H from high to low is in order from DBS group, LBS group, WS group; DBS22H_vs_WS22H_Up, significantly upregulated genes in the DBS group at 22H compare to the WS group; DBS22H > LBS22H > WS22H, genes whose expression level at 22H from high to low is in order from DBS group, LBS group, WS group

The elevated concentrations of Pp IX in shell glands indicates that shell gland plays a crucial role in Pp IX production and eggshell coloring. Therefore, RNA-sequencing (RNA-seq) was performed on 54 shell gland samples from WS, LBS and DBS groups at three time points to identify key genes determining eggshell brownness. After quality control, the number of clean reads produced per sample ranged from 39,733,000 to 55,987,008. At 16H and 22H, the concentration of Pp IX in the shell glands of the DBS groups was much higher than that at 4H; therefore, the DEGs at 16H or 22H versus 4H in DBS group deserve our attention (Fig. 2A and B), and we believe that the regulation of Pp IX formation by gene expression was positive, so the upregulated genes in DBS group shell glands at 16H or 22H compared with 4H were the focus of our analysis. However, the process of Pp IX synthesis, storage, and secretion has a large time overlap with eggshell calcification. In this case, with the WS group that produces white shelled eggs as a control, differentially expressed genes only related to eggshell calcification and not brownness can be identified and separated from those influencing brownness. Using this strategy, 284 upregulated genes at 16H (Gene List 1, Fig. 3A, Additional file 2: Table S1) and 396 upregulated genes at 22H were identified from DBS group shell glands (Gene List 2, Fig. 3B, Additional file 3: Table S2).

At 16H or 22H that belong to eggshell calcification stage, the DEGs between WS group and DBS group deserve attention (Fig. 2C and D). Similarly, we believe that the upregulated genes in shell glands in DBS group are most likely involved in regulating eggshell coloring compared with in WS group. Meanwhile, the eggshell brownness and Pp IX concentration of LBS group was between that of WS and DBS groups, therefore, we applied a criterion to ensure that the gene expression level of LBS group was between WS and DBS groups while screening the significantly expressed genes. Thus, we obtained 312 upregulated genes in the DBS group compared to the WS group at 16H (Gene List 3, Fig. 3C, Additional file 4: Table S3) and 595 upregulated genes at 22H (Gene List 4, Fig. 3D, Additional file 5: Table S4). At 4H, 16H and 22H, there were only 13, 39 and 19 DEGs between LBS and DBS groups, respectively, and these genes were not found to be associated with eggshell brownness.

### Functional annotation of differentially expressed genes

**Fig. 4:**
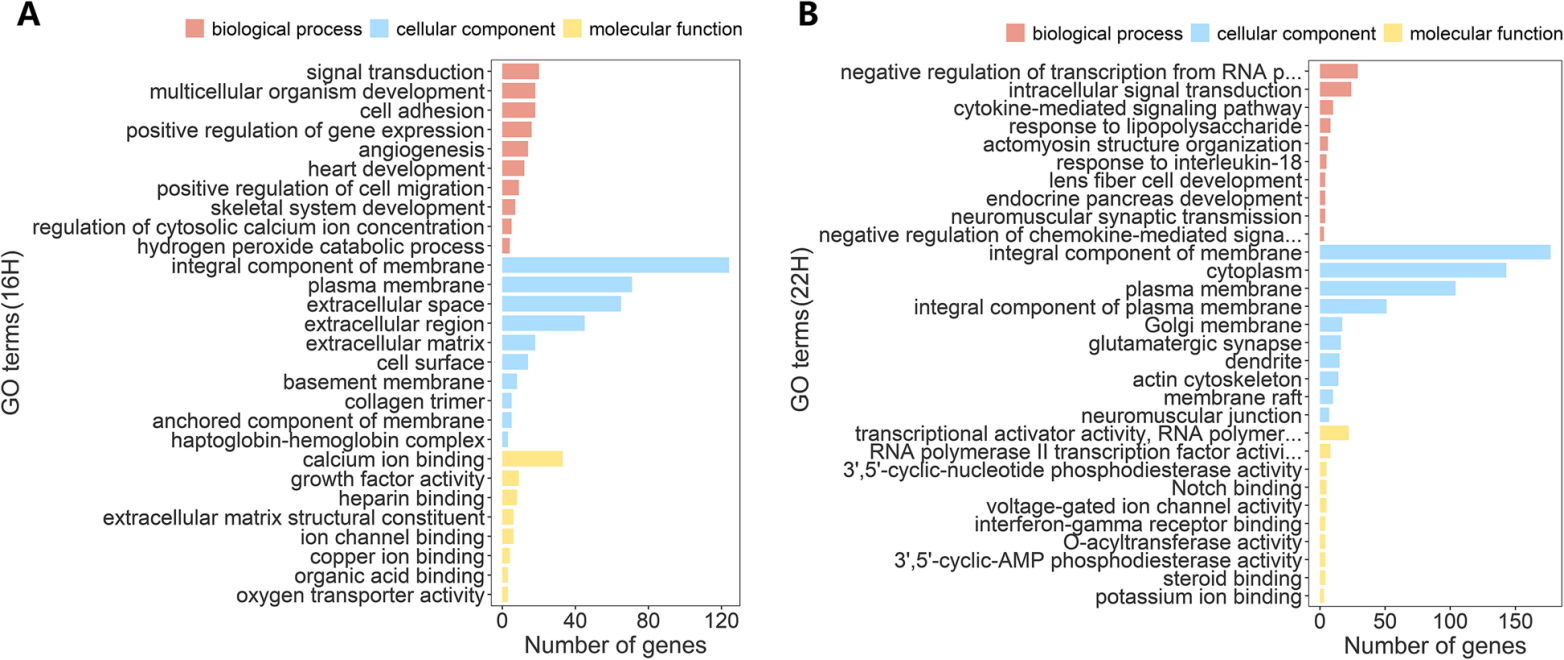
Gene ontology (GO) enrichment analysis. **A** Gene ontology (GO) annotation of upregulated genes at 16H (Gene List 1 and Gene List 3); **B** Gene ontology (GO) annotation of upregulated genes at 22H (Gene List 2 and Gene List 4). The top ten items for biological process, cellular component, and molecular function are shown based on *p*-value

**Fig. 5:**
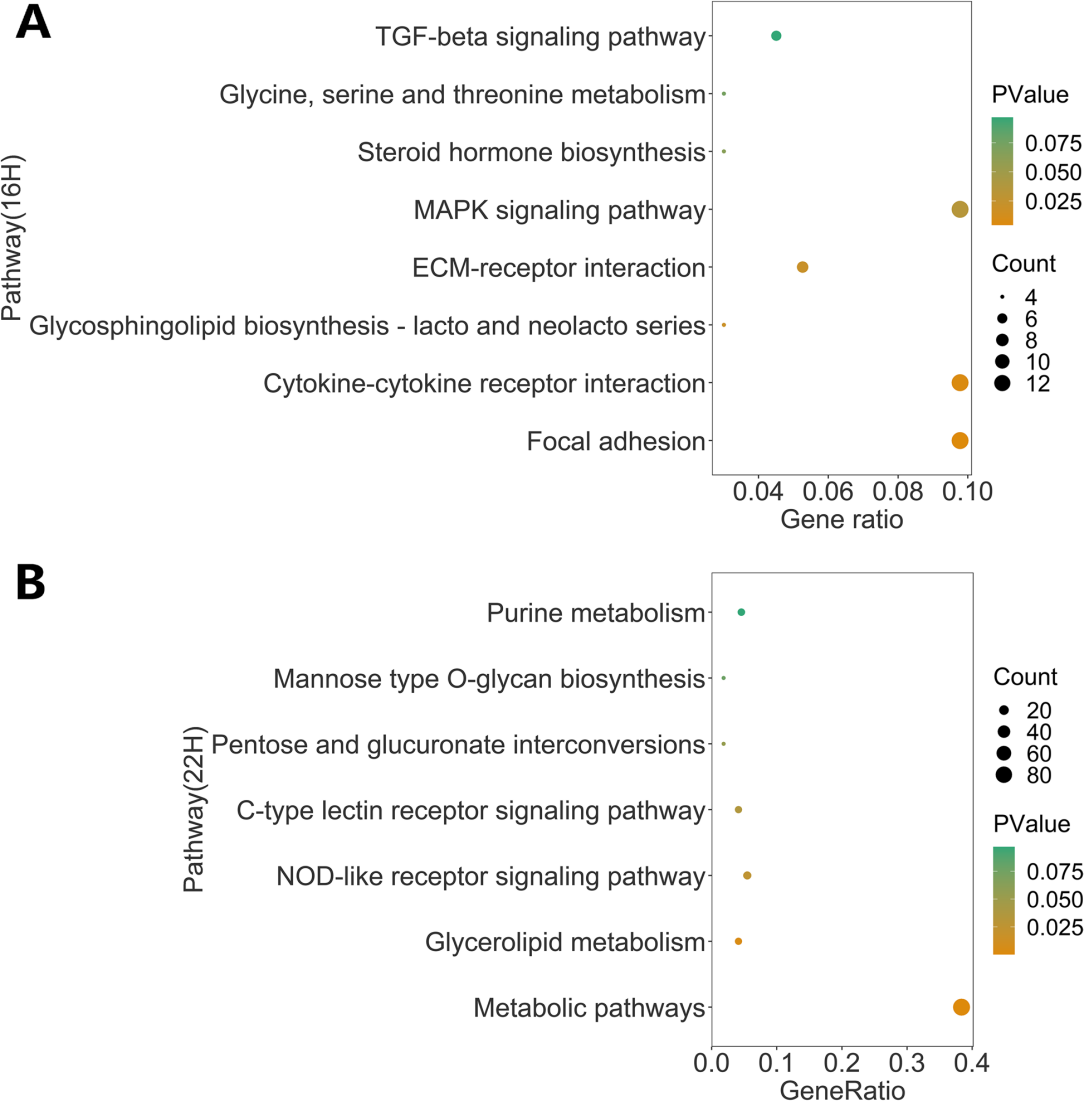
KEGG annotation of upregulated genes. **A** KEGG annotation of upregulated DEGs at 16H (Gene List 1 and Gene List 3). **B** KEGG annotation of upregulated DEGs at 22H (Gene List 2 and Gene List 4)

To verify the biological functions of upregulated DEGs, we merged Gene Lists 1 and 3 (upregulated genes at 16H) and Gene Lists 2 and 4 (upregulated genes at 22H) for GO and KEGG analysis, respectively. For GO enrichment analysis of DEGs at 16H, signal transduction [GO:0007165], integral component of membrane [GO:0016021] and calcium ion binding [GO:0005509] were the top enriched terms for biological process, cellular component, and molecular function, respectively (Gene List 1 and 3) (Fig. 4A). For upregulated genes at 22H (Gene List 2 and 4), negative regulation of transcription from RNA polymerase II promoter [GO:0000122], integral component of membrane [GO:0016021] and transcriptional activator activity, RNA polymerase II transcription regulatory region sequence-specific binding [GO:0001228] were the corresponding top enriched terms, respectively (Fig. 4B). According to the KEGG analysis, upregulated DEGs at 16H were significantly enriched in five pathways including focal adhesion [gga04510], cytokine-cytokine receptor interaction [gga04060], glycosphingolipid biosynthesis - lacto and neolacto series [gga00601], ECM-receptor interaction [gga04512], and the MAPK signaling pathway [gga04010] (*P* < 0.05, Fig. 5A). Metabolic pathways [gga01100], glycerolipid metabolism [gga00561], the NOD-like receptor signaling pathway [gga04621], and the c-type lectin receptor signaling pathway [gga04625] were significantly enriched for upregulated genes at 22H (*P* < 0.05, Fig. 5B).

### Identification of candidate genes

We screened genes for their relation to eggshell pigment from the above DEG lists, wherein *ALAS1* was found in both Gene List 3 and 4. Furthermore, the expression level of *ALAS1* in DBS group was nearly five times that in WS group and nearly twice that in LBS group at 4H. *FLVCR1*, a gene associated with heme export, was found in both Gene List 2 and 4, and its expression in the DBS group was highest at 22H among the three time points in this study. *SLC25A38*, encoding a protein responsible for transporting glycine from the cytoplasm to the mitochondria, was found in Gene List 2. In Gene List 3, we found *ABCG2*, which can transport Pp IX out of the cell, but found no significant difference in the expression level of *ABCG2* at any of the three time points following oviposition in DBS group eggs. Detailed information of these four candidate genes is shown in Table 2.

**Table 2:** Detailed information for the candidate genes responsible for eggshell brownness

Ensembl gene ID	Gene name	Description	Comparison group^a^	Fold change	P adj
ENSGALG00000003948	ALAS1	5′-aminolevulinate synthase 1	DBS16H, WS16H	3.67	1.17E-67
			DBS22H, WS22H	2.19	9.64E-13
ENSGALG00000009807	FLVCR1	Feline leukemia virus subgroup C cellular receptor 1	DBS22H, DBS4H	3.26	8.93E-06
			DBS22H, WS22H	2.15	0.0100
ENSGALG00000041275	SLC25A38	Solute carrier family 25 member 38	DBS22H, DBS4H	2.64	0.0007
ENSGALG00000030677	ABCG2	ATP binding cassette subfamily G member 2	DBS16H, WS16H	2.10	9.72E-06

^a^ DBS16H, hens in DBS group at 16H; WS16H, hens in WS group at 16H; DBS22H, hens in DBS group at 22H; WS22H, hens in WS group at 22H; DBS4H, hens in DBS group at 4H

### Key role of ALAS1 in eggshell brownness verified at transcriptional and genetic levels

**Fig. 6:**
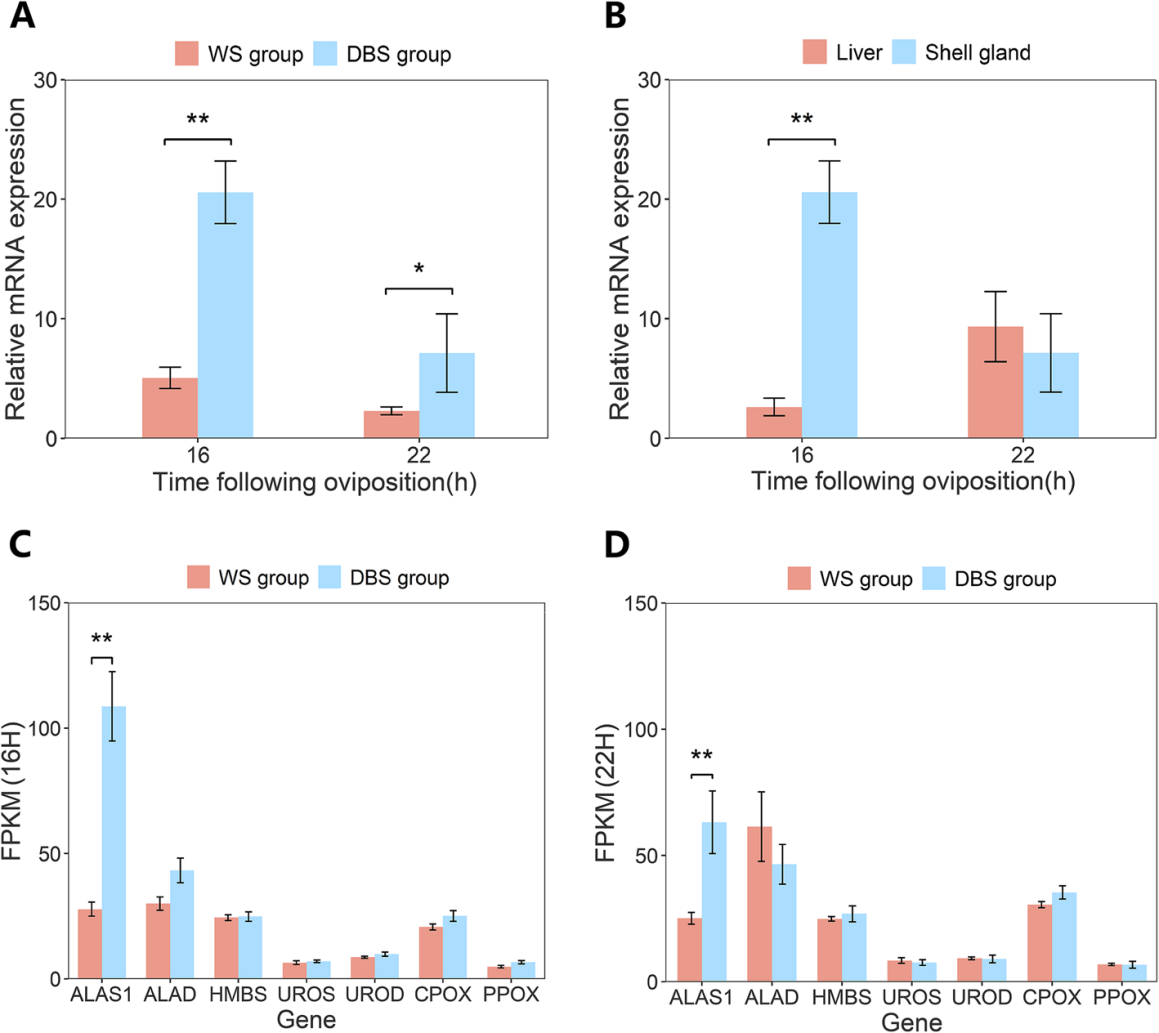
Expression levels of synthase genes in the Pp IX synthesis pathway. **A** *ALAS1* expression levels in the WS group and DBS group at 16H and 22H in the shell gland. **B** *ALAS1* expression levels in the liver and shell gland at 16H and 22H in the DBS group. **C** Expression of enzyme genes in the Pp IX synthesis pathway between WS and DBS groups at 16H in the shell gland. **D** Expression of enzyme genes in the Pp IX synthesis pathway between WS and DBS groups at 22H in the shell gland. *ALAS1*, δ-aminolevulinate synthase 1; *ALAD*, aminolevulinate dehydratase; *HMBS*, hydroxymethylbilane synthase; *UROS*, uroporphyrinogen III synthase; *UROD*, uroporphyrinogen decarboxylase; *CPOX*, coproporphyrinogen oxidase; *PPOX*, protoporphyrinogen oxidase. Data expressed as mean ± SD. **P < 0.01, *P < 0.05

We compared the expression of *ALAS1* in the shell glands of WS group and DBS groups (Fig. 6A), as well as in the shell gland and liver of the DBS group (Fig. 6B). The RT-qPCR results indicated that the expression level of *ALAS1* was significantly higher in the DBS group than in the WS group at 16H and 22H (*P* < 0.01 and *P* < 0.05, Fig. 6A), which was consistent with the RNA-seq results. The expression level of *ALAS1* was significantly higher in the shell gland than in the liver at 16H in the DBS group (*P* < 0.01, Fig. 6B), but there was no significant difference at 22H (*P* > 0.05, Fig. 6B). *ALAS1* is the rate-limiting enzyme in the Pp IX biosynthetic pathway. Thus, we compared the expression of other synthase genes in this pathway between WS and DBS groups based on RNA-seq data, including aminolevulinate dehydratase (*ALAD*), hydroxymethylbilane synthase (*HMBS*), uroporphyrinogen III synthase (*UROS*), uroporphyrinogen decarboxylase (*UROD*), coproporphyrinogen oxidase (*CPOX*), and protoporphyrinogen oxidase (*PPOX*). Results showed that there were no significant differences in gene expression between WS and DBS groups at 16H and 22H except for *ALAS1* (*P* > 0.05, Fig. 6C and D).

To further verify the association between gene expression variations in the *ALAS1* gene and eggshell brownness, the RNA-seq data was used for SNP calling and genotype extraction. Chicken *ALAS1* is located at chromosome 12 and spans 7169 bp. Seven SNPs in *ALAS1* gene were significantly associated with eggshell brownness (*P*_*FDR*_ < 0.05, Table 3). All of the seven SNPs were synonymous mutations, among which four were novel while the other three have already been reported in dbSNP database (https://www.ncbi.nlm.nih.gov/snp/).

**Table 3:** SNPs identified in chicken *ALAS1* DNA sequences

Location	SNPs	Variation type	Region	NCBI number	Genotype^a^		
					WS	LBS	DBS
12:3346427	A → G	Synonymous variant	Exon2	rs734271895	18:0:0	6:9:3	0:6:11
12:3346436	G → A	Synonymous variant	Exon2	–	0:0:18	11:6:1	17:0:0
12:3346451	C → T	Synonymous variant	Exon2	–	18:0:0	6:8:4	0:6:11
12:3347938	G → A	Synonymous variant	Exon4	rs317202842	1:3:14	18:0:0	17:0:0
12:3349200	T → C	Splice region variant, Synonymous variant	Exon6	rs316944705	1:4:13	18:0:0	17:0:0
12:3349584	T → C	Synonymous variant	Exon7	–	0:0:18	10:6:2	17:0:0
12:3349587	G → A	Synonymous variant	Exon7	–	1:4:13	18:0:0	17:0:0

^a^The number ratio of different genotypes in WS, LBS, and DBS groups, number of wild homozygous: number of mutant heterozygous: number of mutant homozygous

### Differential metabolites between LBS and DBS groups

Lots of genes were identified for their differential expression in shell glands of hens laying brown-shell eggs versus white-shell eggs in the current study. However, between light and dark brown eggshell groups, only a few related genes were found at the transcriptome level to account for their pigmented eggshell. Therefore, we utilized metabolomics approaches to investigate metabolites in the shell gland and uterine fluid that may regulate the brownness of eggshells. In the shell gland, 188 and 44 differential metabolites were identified between the LBS and DBS groups at 6H and 22H (Additional files 6 and 7: Tables S5 and S6), respectively. In the uterine fluid, the corresponding numbers of differential metabolites were 84 and 406 (Additional files 8 and 9: Tables S7 and S8), respectively. According to the results of annotation and literature searches, four metabolites were considered as candidate molecules associated with eggshell brownness (Table 4).

**Table 4:** Description of metabolite molecules potentially contributing to eggshell brownness

Metabolites	Comparison group	VIP	Fold change (DBS/LBS)	*p* value
Protoporphyrin IX	shell gland, 6H	1.66	1.64	0.0089
11-hydroxy-E4-neuroprostane	shell gland, 6H	1.76	1.94	0.0091
15-dehydro-prostaglandin E1(1-)	uterine fluid, 22H	1.83	2.02	0.0155
Prostaglandin G2 2-glyceryl Ester	uterine fluid, 22H	1.78	2.02	0.0334

The concentration of Pp IX in shell glands of the DBS group was significantly higher than that in the LBS group at 6H, and we also compared Pp IX concentrations in uterine fluid at 6H and 22H in DBS group and found no significant differences. In addition, 11-hydroxy-E4-neuroprostane was upregulated in shell glands of the DBS group at 6H; 15-dehydro-prostaglandin E1(1-) and prostaglandin G2 2-glyceryl ester were upregulated in uterine fluid of the DBS group at 22H. These three metabolites belong to the class of compounds known as prostaglandins and related compounds.

## Discussion

The subject of whether eggshell color affects eggshell quality has attracted the interest of researchers. In our study, there were no significant differences between LBS and DBS lines in terms of eggshell thickness and eggshell proportion, which is consistent with previous research findings [23, 26]. However, the eggshell strength of DBS line eggs was significantly weaker than that of LBS line eggs, which may be attributed to the influence of eggshell pigment on the eggshell calcification process. Eggshell pigment primarily exists in a vertical crystal layer [27, 28], and can change calcite crystal size and morphology, thus affecting eggshell quality [29]. Pigment particles stored in the shell gland epithelial cells achieve their maximum deposition rate at the end of eggshell calcification [9], which may impair the deposition of vertical crystal layers, resulting in reduced density and a looser structure, thus diminishing eggshell strength.

In this study, Pp IX concentration in the shell glands of the DBS group was significantly higher than that in liver at three time points following oviposition. At 16H and 22H following oviposition, the Pp IX concentration in the shell gland differed significantly across WS, LBS and DBS groups, but not in liver. This confirms that Pp IX is stored in the shell gland before being released into uterine fluid, but does not necessarily imply that Pp IX is synthesized in the shell gland. However, the significantly higher expression of *ALAS1* in the shell gland compared to the liver of the DBS group, as well as its higher expression in shell glands of the DBS group compared to that in the WS group, suggests that Pp IX is synthesized de novo in the shell gland from glycine and succinyl coenzyme A.

As an alternative hypothesis, some scholars posit that Pp IX precursor in the shell gland comes from lysed red blood cells [21, 22]. They suggest that damaged and aging red blood cells are phagocytosed by macrophages to release heme, which is then decomposed by *FECH* into Pp IX. However, in this study, there was no significant difference in the expression level of *FECH* among the three hen groups, indicating that Pp IX in the shell gland is not produced by heme decomposition. This further supports the hypothesis that Pp IX is synthesized de novo in the shell gland.

Although the expression of *ALAS1* in the shell gland differed significantly between WS and DBS groups, we did not find expression differences in other synthase genes in the Pp IX biosynthesis pathway, which may be due to substrate concentrations or transporters responsible for transporting ALA out of the mitochondria. It remains unclear how these genes are regulated. ATP binding cassette subfamily B member 10 (*ABCB10*) is implicated in early mitochondrial steps of heme synthesis, most likely via facilitating ALA export out of the mitochondria [30]. In this study, there was no significant difference in *ABCB10* expression among the three groups at any of the three post-oviposition time points. In addition, research has demonstrated that *SLC25A38* can promote ALA production by delivering glycine into the mitochondria or by exchanging glycine through the inner mitochondrial membrane of red blood cells [31]. Our results show that the expression of *SLC25A38* in the DBS group was significantly higher than that in the WS group at 22H, suggesting that the synthesis of Pp IX may still be taking place in the shell gland epithelial cells during rapid deposition of Pp IX. It remains to be determined whether *SLC25A38* in the epithelial cells of the shell gland play the same role as in red blood cells.

**Fig. 7:**
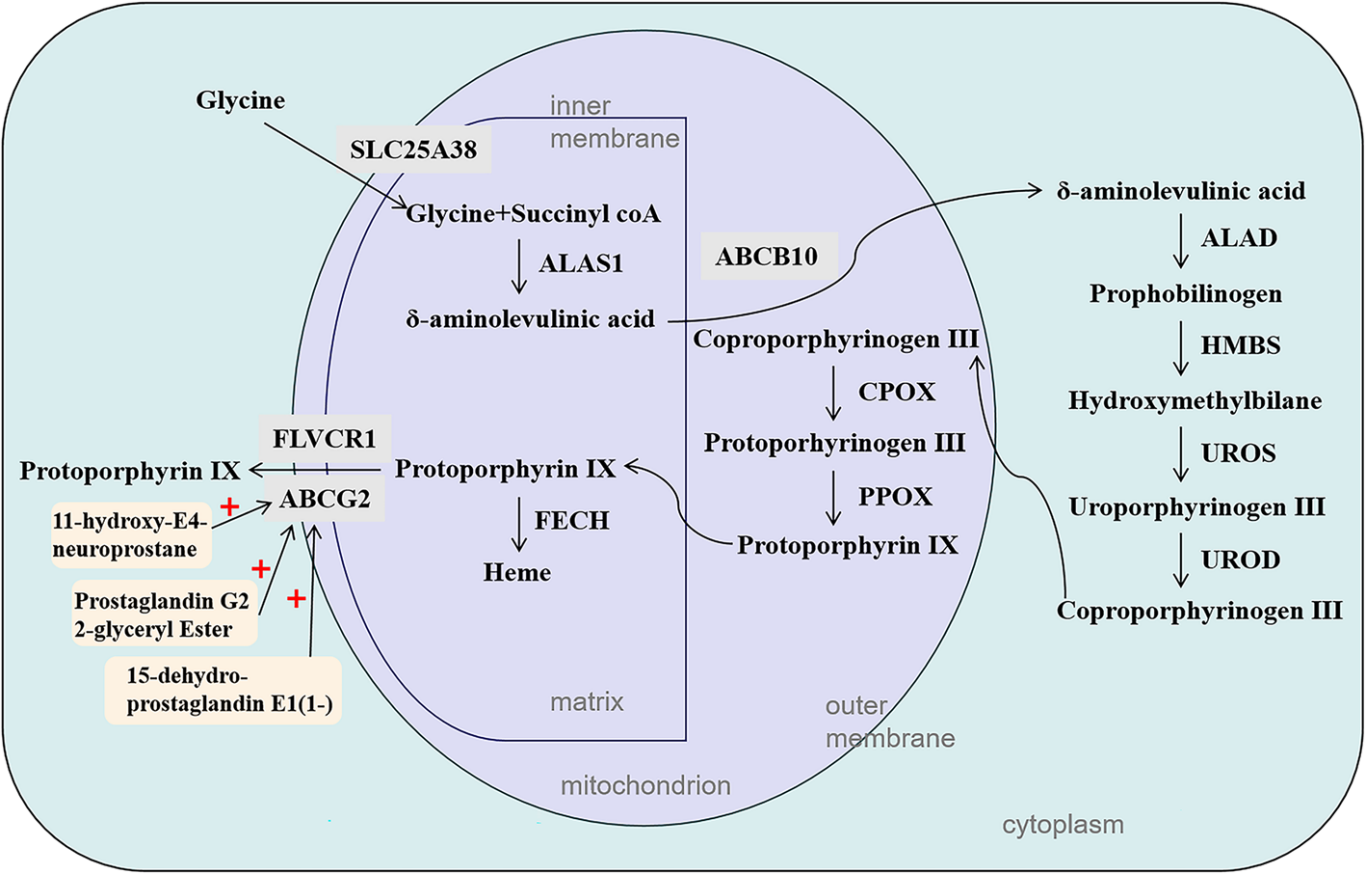
Schematic representation of protoporphyrin IX/heme biosynthetic pathway. *ALAS1*, δ-aminolevulinate synthase 1; *ALAD*, aminolevulinate dehydratase; *HMBS*, hydroxymethylbilane synthase; *UROS*, uroporphyrinogen III synthase; *UROD*, uroporphyrinogen decarboxylase; *CPOX*, coproporphyrinogen oxidase; *PPOX*, protoporphyrinogen oxidase; *FECH*, ferrochelatase. The red plus sign indicates activation

All genes in the Pp IX synthesis pathway were expressed in the WS group, however, their eggshells remain colorless. We hypothesize that a potential cause is the low expression level of *ALAS1* in the WS group eggs, resulting in low Pp IX production, which is almost entirely utilized for the synthesis of heme. Another possibility is that the Pp IX outflow route from the shell gland to uterine fluid may be restricted. *ABCG2* is a member of the ATP-binding cassette family and is essential for the export of Pp IX from the cell [24, 32, 33]. At 16H, the expression level of *ABCG2* was significantly higher in the DBS group compared to the WS group, but did not alter significantly along these three time points following oviposition, consistent with the results of previous studies [19]. So dose *ABCG2* have the function of exporting Pp IX from the shell gland epithelium? There was no significant difference in the concentration of Pp IX in the uterine fluid in DBS group between 6H and 22H, and 22H is the stage when Pp IX is deposited on the eggshell surface, indicating that Pp IX has been gradually released into the uterine fluid since 6H. This means that the transporter proteins that transport Pp IX out of the shell gland epithelial cells have been expressed since 6H. And why Pp IX is sustained release, but only the surface of the shell appears brown? We speculate that during the prophase and metaphase of eggshell mineralization, Pp IX transportation is in trace amounts, so it’s not macroscopic. Study has shown that eggshell pigment is distributed in all layers including the shell membrane, but the amount of deposition varies greatly [34]. At 16H, the concentration of Pp IX in shell gland of DBS group was significantly higher than that at 4H, indicating that the synthesis rate of Pp IX in shell gland was greater than the release rate, and Pp IX accumulated continuously in shell gland. Intrauterine injection of prostaglandins has been shown to induce pigment secretion [35]. We speculate that when calcification is almost complete，*ABCG2* or other unknown transporters are stimulated by certain factors, such as threshold Pp IX concentration or prostaglandins, and their gene expression or protein activity is activated, resulting in rapid release of Pp IX from shell glands in large quantities and deposition on the eggshell surface. In this study, the transcript level of *ABCG2* was not significantly increased at 22H, so we speculated that the translation process or protein activity of *ABCG2* might be activated，which needs to be further studied. In addition, *FLVCR1* has been thought to encode a protein for exporting heme [36], but prior studies have shown that *FLVCR1* can export endogenous synthetic Pp IX when ALA or iron is present [37]. We discovered that the expression level of *FLVCR1* in DBS group was significantly higher compared to WS group at 22H and was highest at 22H compared to 4H and 16H. It is noteworthy that *FLVCR1* may be able to export Pp IX synthesized in shell glands. It is the combined interactions of these genes and metabolites that leads to variation in eggshell brownness (Fig. 7).

The expression level of *ALAS1* was found to be closely related to the intensity of eggshell brownness. Seven SNPs in *ALAS1* gene are synonymous mutations, which do not change the sequence of amino acids. The replacement of synonymous codons, however, may change the synthesis rate of polypeptide chain and affect protein folding and conformation, thus affecting the function of protein [38, 39]. Therefore, SNPs of the *ALAS1* may regulate the expression of the ALAS1 gene, thereby affecting eggshell brownness.

GO and KEGG functional enrichment analysis also provided us with information regarding eggshell deposition and pigmentation. At 16H, signal transduction pathways were highly enriched; at 22H, signal transduction was also prevalent and regulation of transcriptional processes was active, indicating that the expression of genes associated with eggshell and eggshell color deposition in the shell gland epithelial cells was stably regulated and maintained, possibly via the membrane. There may be a connection between lipid metabolism and eggshell deposition and pigmentation, which warrants further study.

## Conclusions

The content of Pp IX in the shell gland varies with eggshell color. Eggshell pigment Pp IX is synthesized de novo and stored in the shell gland. *ALAS1* plays a key role in the Pp IX pathway and can influence eggshell brownness by influencing Pp IX production in the shell gland. Transporters such as *SLC25A38*, *ABCG2* and *FLVCR1* and metabolites such as 11-hydroxy-E4-neuroprostane, 15-Dehydroprostaglandin E1(1-) and Prostaglandin G2 2-glyceryl ester may regulate eggshell brownness. According to the results of our study, we think that ALAS1 gene should be knocked down in brown-shell laying hens next to verify its critical role in eggshell brownness, and proteomic studies that can reflect the translation level are also essential. This study lays a foundation for studying the formation mechanisms of brown eggshell color. These results may contribute to further research on the mechanism of eggshell browning.

## Materials and methods

### Animal management and sample collection

A chicken population that was derived from the Rhode Island Red line were bidirectionally selected for six generations according to their eggshell brownness and resulted in two experimental lines with light brown eggshells (LBS) and dark brown eggshells (DBS). These two lines were raised in individual cages and allowed free access to food and water. The temperature of the room was controlled at 22 °C, and the daily lighting plan was 16 L:8D. At 54 weeks of age, 150 laying hens were randomly selected from LBS and DBS lines respectively, and egg quality was measured on the same day. Eighteen White Leghorn hens, 35 LBS line hens, and 35 dB line hens were selected at 57 weeks of age, with regular laying time and uniform eggshell color, and were assigned to the white eggshell (WS) group, light brown eggshell (LBS) group, and dark brown eggshell (DBS) groups, respectively. A total of 54 hens (18 hens per group) were euthanized — six hens per group were euthanized at 4-, 16- and 22-hour timepoints following oviposition. The location of the next ovulated egg in the oviduct at different time points following oviposition is shown in Additional file 10: Fig. S2. Liver and mucosal layer of shell gland samples (108 total) were collected and stored at − 80 °C for Pp IX concentration determination and RNA extraction. The remaining 34 hens in LBS and DBS groups were euthanized — eight hens were euthanized at 6 h following oviposition, while nine were euthanized at 22 h following oviposition in each group, and mucosal layer of shell gland and uterine fluid samples (in total of 68) were collected and stored at − 80 °C for subsequent ultra-high performance liquid chromatography - tandem mass spectrometry (UHPLC-MS/MS) analysis. Shell glands and Uterine fluid were collected as follows: at 6 h following oviposition, the oviduct was cut off at the vaginal site, and the shell gland was carefully cut longitudinally though the vaginal opening to expose its mucosal layer. The egg with a very thin shell was removed, and the uterine fluid on the mucosa surface of the shell gland was repeatedly aspirated with a syringe. At 22 h following oviposition, we cut off the oviduct at the vaginal site, extruded the egg in the shell gland through the vaginal site, and extruded the shell gland from the top down to extrude the uterine fluid through the vaginal opening. After collecting the uterine fluid, we cut out the entire shell gland and rinsed it with normal saline, then gently scraped the mucosal layer of the shell gland with the handle of a small medicine spoon.

### Eggshell quality determination

Egg weight, L, a, and b values, eggshell strength, eggshell thickness, eggshell weight, and eggshell proportion were determined for 150 eggs of LBS line and DBS line respectively. Egg weight and eggshell weight were measured using an electronic balance. The values of L, a, and b were determined at the center of sharp end, equator, and blunt end by a reflectometer (Minolta CM600). An egg force reader (EMG-0502, Robotmation, Japan) was used to measure eggshell strength. Eggshell thickness was measured at the sharp end, equator, and blunt end of eggs using a micrometer screw gauge (Mitutoyo293-100, Kawasaki, Japan). Eggshell proportion was the ratio of the weight of the eggshell to the weight of the egg.

### Pp IX concentration determination

The extraction solution was prepared as described previously [18], which consisted of hydrochloric acid, acetonitrile, and water. The shell gland and the liver were ground to powders in liquid nitrogen. Prepared shell gland and liver powders (both 0.25 g) were vortexed with 1.5 mL extraction solution in a centrifuge tube for 15 seconds. Dissolved samples were centrifuged for 10 min at 17,800 × *ɡ* at 25 °C, and 0.3 mL supernatant was transferred to a microtiter plate for analysis using a microplate reader (SpectraMax i3x, Meigu Molecular Instrument Co., Ltd., Austria) at the wavelength of 412 nm. Pp IX standard curve was established to calculate the Pp IX concentration of the measured samples.

### RNA extraction, library construction, and transcriptome sequencing

Total RNA from 54 shell gland samples was isolated using TRIzol reagent [40] (Invitrogen, CA, USA) following the manufacturer’s protocol. RNA Nano 6000 Assay Kit of the Bioanalyzer 2100 system (Agilent Technologies, CA, USA) was used to accurately detect RNA integrity [41]. The initial amount RNA for library construction was 1 μg. The mRNA with polyA tail was enriched by poly-T oligo-attached magnetic beads [42]. The obtained mRNA was then randomly fragmented with divalent cations in Fragmentation Buffer at high temperature. The first strand of cDNA was synthesized in the M-MuLV Reverse Transcriptase system using mRNA fragments as templates and random oligonucleotides as primers, then the RNA strand was degraded with RNase H, and the second strand of cDNA was synthesized from dNTPs in the DNA polymerase I system. Subsequently, the end of the double-stranded cDNA was repaired. After adenylation at 3′ ends of DNA fragments, Adaptor with hairpin loop structure were ligated to prepare for hybridization. AMPure XP system (Beckman Coulter, Beverly, USA) was used to screen 370-420 bp cDNA fragments, then PCR was performed. At last, PCR products were purified by AMPure XP system, and the library quality was evaluated by Agilent Bioanalyzer 2100 system [43]. TruSeq PE Cluster Kit V3-CBOT-HS (Illumia) was used to cluster the index-coded samples on a cBot Cluster Generation System. Following cluster generation, library preparation was sequenced on Illumina Novaseq platform to generate 150 bp paired-end reads.

### Expression quantification and differentially expressed gene analysis

Raw data of fastq format were firstly processed through Perl scripts which removed reads with adapter, reads with ploy-N and low-quality reads from raw data to obtain the clean data. Clean reads were aligned to Genome Reference Consortium Chicken Build 6a (GRCg6a) assembly provided by Ensembl (https://asia.ensembl.org/index.html) using HISAT (ver 2-2.1.1). The mapped reads of each sample were assembled and quantified by StringTie (ver 2.1.4).

DEGs were identified using the DEseq2 Bioconductor package based on the generalized linear model. The screening criteria for differentially expressed genes was set at *p*-value < 0.05, |Log_2_FC| > 1. The online biological tool DAVID (https://david.ncifcrf.gov/) was used for gene ontology (GO) and Kyoto encyclopedia of genes and genomes (KEGG) [44–46] pathway enrichment analysis of the DEGs. A value of *P* < 0.05 was considered to have statistical significance.

### Quantitative real-time polymerase chain reaction analysis

Total RNA was extracted from the shell gland and the liver with Eastep® Super Total RNA Extraction Kit (Promega, Shanghai, China). All operations were performed at 4 °C or on ice following the manufacturer’s instructions. Briefly, 1 μg of total RNA was used for cDNA synthesis using PrimeScript™ RT reagent Kit (Takara, Japan). RT-qPCR was performed using cDNA with CFX96 Real-Time PCR Detection System (Bio-Rad, USA) and all samples were run in triplicate using TB Green® Premix Ex Taq™ (Takara, Japan). The primer sequences were as follows (5′ to 3′): β-actin, forward: TATGTGCAAGGCCGGTTTC, reverse: TGTCTTTCTGGCCCATACCAA [18]; *ALAS1*, forward: GGTGGACAGGAAAGGTAAAGA, reverse: ACTGGTCATACTGGAAGGTG [18]. The RT-qPCR procedure was 95 °C for 30 s, 40 cycles of 95 °C for 5 s and 60 °C for 35 s.

### SNPs calling with transcriptome data

Clean data of the same eggshell color were combined into one group and divided into WS group, LBS group and DBS group. We mapped reads to GRCg6a assembly with STAR (ver 2.7.9a). Repeated reads were removed using GATK (ver 4-4.2.3.0). SNP detection was performed using GATK’s HaplotypeCaller. Dual alleles were extracted using GATK’s SelectVariants. Variable filtration was used for strict quality control of SNP detected, with the following criteria: 1) QD < 2.0; 2) QUAL < 30.0; 3) MQ < 40.0; 4) FS > 60.0; 5) MQRankSum < − 12.5; 6) ReadPosRankSum < − 8.0. Vcftools (ver 0.1.16) [47] was used to conduct quality control on SNP data sets. The quality control criteria were: 1) Sample detection rate > 90%; 2) Minimum allele frequency > 5%. Then Beagle (ver 5.2) [48] was used for gefnotype filling. PLINK (ver 1.90) was used for case-control association analysis and extracted genotypes of significant SNP contained in candidate gene sequences. Finally, SNPS are annotated based on GRCg6a assembly.

As the traditional Bonferroni correction is too conservative [49], simpleM package [50] was used to calculate the number of valid independent tests In this study, the number of valid independent tests was 2200, so at the genome-wide significance level, the threshold *p*-value was set as 2.27E-05.

### Metabolite extraction

Shell gland tissues (100 mg) were ground separately with liquid nitrogen. The homogenate was vortexed with precooled 80% methanol. Then the mixtures were centrifuged at 15000 × *ɡ*, 4 °C for 20 min after placed on ice for 5 min. The methanol concentration in supernatant was reduced to 53% by adding LC-MS grade water. Then the samples were centrifuged at 15000 × ɡ, 4 °C for 20 min after moved to a Eppendorf tube. In the end, the obtained supernatant was used for LC-MS/MS analysis [51].

Uterine fluids (100 μL) were individually transferred to the Eppendorf tubes and vortexed with precooled 80% methanol. The following procedure was the same as described above for the shell gland part. In the end, the obtained supernatant was used for LC-MS/MS analysis [52, 53].

### UHPLC-MS/MS analysis

UHPLC-MS/MS analyses were performed using a Vanquish UHPLC system (ThermoFisher, Germany) coupled with an Orbitrap Q Exactive™ HF mass spectrometer (Thermo Fisher, Germany). A 17-min linear gradient with a flow rate of 0.2 mL/min was used to infuse samples into a Hypesil Goldcolumn (100 × 2.1 mm, 1.9 μm). The eluents in positive pole mode were 0.1% formic acid aqueous solution (A) and methanol (B) as well as those in negative pole mode were 5 mM ammonium acetate, pH 9.0(A) and methanol (B). The solvent gradient was set as follows: 2% B, 1.5 min; 2-100% B, 3 min; 100% B, 10 min; 100-2% B, 10.1 min; 2% B, 12 min. The mass spectral data were obtained from Q ExactiveTM mass spectrometer carrying out in positive/negative pole mode, and the parameters included spray voltage of 3.5 kV, capillary temperature of 320 °C, sheath gas flow rate of 35 psi and aux gas flow rate of 10 L/min, S-lens RF level of 60, Aux gas heater temperature of 350 °C.

### Identification and quantification of metabolites

The raw data were processed using The Compound Discoverer 3.1 (CD3.1, ThermoFisher) for peak alignment, peak picking and quantitation. Peak intensities were normalized to the total spectral intensity, and the molecular formula was predicted based on additive ions, molecular ion peaks and fragment ions. Whereafter, mzCloud (https://www.mzcloud.org/), mzVault and MassList database were used for peak matching. At last, accurate qualitative and relative quantitative results were obtained. OPLS-DA and t-test were used to analyze the expression of metabolites. The screening criteria for differential metabolites were VIP > 1, *P* < 0.05, and |Log_1.5_FC| > 1. KEGG pathway enrichment analysis of differential metabolites was performed using MetaboAnalyst (https://www.metaboanalyst.ca/) online analysis platform. A value of *P* < 0.05 was considered to have statistical significance.

### Statistical analysis

Statistical analysis was performed with R software (ver 4.1.1). The eggshell quality between LBS line and DBS line, Pp IX concentrations between liver and shell glands, the gene expression levels between WS group and DBS group as well as between the liver and shell gland were analyzed by t-test. The eggshell quality, the Pp IX concentrations among WS, LBS, and DBS groups were analyzed by analysis of variance. Differences were considered statistically significant at *P* < 0.05.

## Supplementary Information

**Additional file 1: Fig. S1.** Eggs in shell glands of WS, LBS, and DBS groups at 16 hours and 22 hours following oviposition.

**Additional file 2: Table S1.** Differentially upregulated genes in DBS group at 16 h compared with 4 h following oviposition.

**Additional file 3: Table S2.** Differentially upregulated genes in DBS group at 22 h compared with 4 h following oviposition.

**Additional file 4: Table S3.** Differentially upregulated genes in DBS group compared with WS group at 16 h following oviposition.

**Additional file 5: Table S4.** Differentially upregulated genes in DBS group compared with WS group at 22 h following oviposition.

**Additional file 6: Table S5.** Differential metabolites between LBS and DBS groups at 6 h following oviposition in shell glands.

**Additional file 7: Table S6.** Differential metabolites between LBS and DBS groups at 22 h following oviposition in shell glands.

**Additional file 8: Table S7.** Differential metabolites between LBS and DBS groups at 6 h following oviposition in uterine fluid.

**Additional file 9: Table S8.** Differential metabolites between LBS and DBS groups at 22 h following oviposition in uterine fluid.

**Additional file 10: Fig. S2.** The location of the next ovulated egg in the oviduct at different time points following oviposition.

## Data Availability

The raw data are available from the BioProject database with accession number PRJNA871302. (Reviewer link: https://dataview.ncbi.nlm.nih.gov/object/PRJNA871302?reviewer=nkrvnijcf1tvmij6tefa1b99eo).

## Abbreviations

Pp IX: Protoporphyrin IX
ALAS1: δ-aminolevulinate synthase 1
SLC25A38: Solute carrier family 25, member 38
ABCG2: ATP binding cassette subfamily G member 2
FLVCR1: Feline leukemia virus subgroup C cellular receptor 1
DEGs: Differentially expressed genes
SNPs: Single nucleotide polymorphisms
ALA: δ-aminolevulinic acid
FECH: Ferrochelatase
CPOX: Coproporphyrinogen oxidase
LBS: Light brown eggshell
DBS: Dark brown eggshell
4H: 4 hours following oviposition
16H: 16 hours following oviposition
22H: 22 hours following oviposition
WS: White eggshell
RNA-seq: RNA-sequencing
ABCB10: ATP binding cassette subfamily B member 10
UHPLC-MS/MS: Ultra-high performance liquid chromatography - tandem mass spectrometry
GRCg6a: Genome Reference Consortium Chicken Build 6a
GO: Gene ontology
KEGG: Kyoto encyclopedia of genes and genomes
ALAD: Aminolevulinate dehydratase
HMBS: Hydroxymethylbilane synthase
UROS: Uroporphyrinogen III synthase
UROD: Uroporphyrinogen decarboxylase
CPOX: Coproporphyrinogen oxidase
PPOX: Protoporphyrinogen oxidase

## References

[1] Zhang LC, Ning ZH, Xu GY, Hou ZC, Yang N (2005). Heritabilities and genetic and phenotypic correlations of egg quality traits in brown-egg dwarf layers. Poult Sci.

[2] Drabik K, Batkowska J, Vasiukov K, Pluta A (2020). The Impact of Eggshell Colour on the Quality of Table and Hatching Eggs Derived from Japanese Quail. Animals.

[3] Lan LTT, Nhan NTH, Hung LT, Diep TH, Xuan NH, Loc HT, Ngu NT (2021). Relationship between plumage color and eggshell patterns with egg production and egg quality traits of Japanese quails. Vet World.

[4] Yang H, Wang Z, Jian LU, Ping HU, Wang JJ (2008). The Relationship Between Eggshell Color and Egg Quality as well as Eggshell Ultrastructure.

[5] Kennedy GY, Vevers HG (1976). A survey of avian eggshell pigments. Comp Biochem Physiol B.

[6] Miksik I, Holáň V, Deyl Z (1994). Quantification and variability of eggshell pigment content. Comp Biochem Physiol Physiol.

[7] Lu MY, Xu L, Qi GH, Zhang HJ, Wu SG (2021). Mechanisms Associated with the Depigmentation of Brown Eggshells: A Review. Poult Sci.

[8] Fernandez MS, Escobar C, Lavelin I, Pines M, Arias JL (2003). Localization of osteopontin in oviduct tissue and eggshell during different stages of the avian egg laying cycle. J Struct Biol.

[9] Tamura T, Fujii S (1966). Histological observations on the quail oviduct ; on the secretions in the mucous epithelium of the uterus. J Faculty FisheriesAnimal Husbandry Hiroshima Univ.

[10] Nys Y, Zawadzki J, Gautron J, Mills AD (1991). Whitening of brown-shelled eggs: mineral composition of uterine fluid and rate of protoporphyrin deposition. Poult Sci.

[11] Wolford JH, Ringer RK, Coleman TH (1964). Ovulation and Egg Formation in the Beltsville Small White Turkey. Poult Sci.

[12] Sachar M, Anderson KE, Ma X (2016). Protoporphyrin IX: the Good, the Bad, and the Ugly. J Pharmacol Exp Ther.

[13] May BK, Dogra SC, Sadlon TJ, Bhasker CR, Cox TC, Bottomley SS (1995). Molecular Regulation of Heme Biosynthesis in Higher Vertebrates. Prog Nucleic Acid Res Mol Biol.

[14] Gibson KD, Laver WG. Neuberger A. Initial stages in the biosynthesis of porphyrins. 2. The formation of δ-aminolaevulic acid from glycine and succinyl-coenzyme A by particles from chicken erythrocytes. Biochem J. 1958;70(1):71–81. 10.1042/bj0700071. 10.1042/bj0700071 PMC1196628 13584304

[15] Phillips DH, Kushner JP, Ajioka RS (2006). Biosynthesis of heme in mammals. Biochim Biophys Acta.

[16] Schwartz S, Raux WA, Schacter BA, Stephenson BD, Shoffner RN (1980). Loss of hereditary uterine protoporphyria through chromosomal rearrangement in mutant Rhode Island Red hens. Int J Biochem.

[17] Hargitai R, Boross N, Hámori S, Neuberger E, Nyiri ZJP, Pbz BZ (2017). Eggshell Biliverdin and Protoporphyrin Pigments in a Songbird: Are They Derived from Erythrocytes, Blood Plasma, or the Shell Gland?. Physiol Biochem Zool.

[18] Li G, Chen S, Duan Z, Qu L, Xu G, Yang NJPS (2013). Comparison of protoporphyrin IX content and related gene expression in the tissues of chickens laying brown-shelled eggs.

[19] Samiullah S, Roberts J, Wu SB (2017). Downregulation of ALAS1 by nicarbazin treatment underlies the reduced synthesis of protoporphyrin IX in shell gland of laying hens. Sci Rep.

[20] Keneddy GY, Vevers HG (1973). Eggshell pigments of the araucano fowl. Comp Biochem Physiol B.

[21] De Coster G, De Neve L, Lens L (2012). Intraclutch variation in avian eggshell pigmentation: the anaemia hypothesis. Oecologia.

[22] Wang XT, Zhao CJ, Li JY, Xu GY, Lian LS, Wu CX, Deng XM (2009). Comparison of the total amount of eggshell pigments in Dongxiang brown-shelled eggs and Dongxiang blue-shelled eggs. Poult Sci.

[23] Lu MY, Wang WW, Qi GH, Xu L, Wang J (2020). Mitochondrial transcription factor A induces the declined mitochondrial biogenesis correlative with depigmentation of brown eggshell in aged laying hens. Poult Sci.

[24] Kobuchi H, Moriya K, Ogino T, Fujita H, Utsumi TJPO (2012). Mitochondrial Localization of ABC Transporter ABCG2 and Its Function in 5-Aminolevulinic Acid-Mediated Protoporphyrin IX Accumulation. PLoS One.

[25] Zheng C, Li Z, Yang N, Ning Z (2014). Quantitative expression of candidate genes affecting eggshell color. Anim Sci J.

[26] Guangqi L, Congjiao S, Guiqin W, Fengying S, Aiqiao L, Ning Y, Gomez-Casati DF (2016). iTRAQ-based quantitative proteomics identifies potential regulatory proteins involved in chicken eggshell brownness. PLoS One.

[27] Samiullah S, Roberts JR (2013). The location of protoporphyrin in the eggshell of brown-shelled eggs. Poult Sci.

[28] Bain MM, Mcdade K, Burchmore R, Law A, Wilson PW, Schmutz M, Preisinger R, Dunn IC (2013). Enhancing the egg's natural defence against bacterial penetration by increasing cuticle deposition. Anim Genet.

[29] Wang X, Zhu P, Sun Z, Zhang J, Sun C (2021). Uterine Metabolomic Analysis for the Regulation of Eggshell Calcification in Chickens. Metabolites.

[30] Bayeva M, Khechaduri A, Wu R, Burke MA, Wasserstrom JA, Singh N, Liesa M, Shirihai OS, Langer NB, Paw BH (2013). ABCB10 Regulates Early Steps of Heme Synthesis. Circ Res.

[31] Guernsey DL, Jiang H, Campagna DR, Evans SC, Ferguson M, Kellogg M, Lachance M, Matsuoka M, Nightingale M, Rideout A (2009). Mutations in mitochondrial carrier family gene SLC25A38 cause nonsyndromic autosomal recessive congenital sideroblastic anemia. Nat Genet.

[32] Zhou S, Zong Y, Ney PA, Nair G, Sorrentino BP (2005). Increased expression of the Abcg2 transporter during erythroid maturation plays a role in decreasing cellular protoporphyrin IX levels. Blood.

[33] Ogino T, Kobuchi H, Munetomo K, Fujita H, Yamamoto M, Utsumi T, Inoue K, Shuin T, Sasaki J, Inoue M (2011). Serum-dependent export of protoporphyrin IX by ATP-binding cassette transporter G2 in T24 cells. Mol Cell Biochem.

[34] Wang XT, Deng XM, Zhao CJ, Li JY, Xu GY, Lian LS, Wu CX (2007). Study of the deposition process of eggshell pigments using an improved dissolution method. Poult Sci.

[35] Soh T, Koga O (1999). Effects of phosphate, prostaglandins, arachidonic acid and arginine vasotocin on oviposition and pigment secretion from the shell gland in Japanese quail. Br Poult Sci.

[36] Quigley JG, Yang Z, Worthington MT, Phillips JD, Sabo KM, Sabath DE, Berg CL, Sassa S, Wood BL, Abkowitz JL (2004). Identification of a human heme exporter that is essential for erythropoiesis. Cell.

[37] Yang Z, Philips JD, Doty RT, Giraudi P, Ostrow JD, Tiribelli C, Smith A, Abkowitz JL (2010). Kinetics and specificity of feline leukemia virus subgroup C receptor (FLVCR) export function and its dependence on hemopexin. J Biol Chem.

[38] Kimchi-Sarfaty C, Oh JM, Kim IW, Sauna ZE, Calcagno AM, Ambudkar SV, Gottesman MM (2007). A “silent” polymorphism in the MDR1 gene changes substrate specificity. Science (New York, NY).

[39] Walsh IM, Bowman MA, Soto Santarriaga IF, Rodriguez A, Clark PL (2020). Synonymous codon substitutions perturb cotranslational protein folding in vivo and impair cell fitness. Proc Natl Acad Sci U S A.

[40] Rio DC, Ares M, Hannon GJ, Nilsen TW (2010). Purification of RNA using TRIzol (TRI reagent). Cold Spring Harb Protoc.

[41] Popova M, Martin C, Morgavi DP (2010). Improved protocol for high-quality co-extraction of DNA and RNA from rumen digesta. Folia Microbiol.

[42] Bachman J (2013). Reverse-transcription PCR (RT-PCR). Methods Enzymol.

[43] Modi A, Vai S, Caramelli D, Lari M (2021). The Illumina Sequencing Protocol and the NovaSeq 6000 System. Methods Mol Biology (Clifton, NJ).

[44] Kanehisa M, Goto S (2000). KEGG: kyoto encyclopedia of genes and genomes. Nucleic Acids Res.

[45] Kanehisa M, Furumichi M, Sato Y, Ishiguro-Watanabe M, Tanabe M (2021). KEGG: integrating viruses and cellular organisms. Nucleic Acids Res.

[46] Kanehisa M, Sato Y, Kawashima M, Furumichi M, Tanabe M (2016). KEGG as a reference resource for gene and protein annotation. Nucleic Acids Res.

[47] Purcell S, Neale B, Todd-Brown K, Thomas L, Ferreira M, Bender D, Maller J, Sklar P, Bakker P, Daly MJ (2007). PLINK: a tool set for whole-genome association and population-based linkage analyses. Am J Hum Genet.

[48] Browning SR, Browning BL (2007). Rapid and accurate haplotype phasing and missing-data inference for whole-genome association studies by use of localized haplotype clustering. Am J Hum Genet.

[49] Bland JM, Altman DG (1995). Multiple significance tests: the Bonferroni method. BMJ.

[50] Gao X (2011). Multiple testing corrections for imputed SNPs. Genet Epidemiol.

[51] Want EJ, Masson P, Michopoulos F, Wilson ID, Theodoridis G, Plumb RS, Shockcor J, Loftus N, Holmes E, Nicholson JK (2013). Global metabolic profiling of animal and human tissues via UPLC-MS. Nat Protoc.

[52] Want EJ, O'Maille G, Smith CA, Brandon TR, Uritboonthai W, Qin C, Trauger SA, Siuzdak G (2006). Solvent-dependent metabolite distribution, clustering, and protein extraction for serum profiling with mass spectrometry. Anal Chem.

[53] Barri T, Dragsted LO (2013). UPLC-ESI-QTOF/MS and multivariate data analysis for blood plasma and serum metabolomics: effect of experimental artefacts and anticoagulant. Anal Chim Acta.

